# MGEx-Udb: A Mammalian Uterus Database for Expression-Based Cataloguing of Genes across Conditions, Including Endometriosis and Cervical Cancer

**DOI:** 10.1371/journal.pone.0036776

**Published:** 2012-05-11

**Authors:** Akhilesh K. Bajpai, Sravanthi Davuluri, Darshan S. Chandrashekar, Selvarajan Ilakya, Mahalakshmi Dinakaran, Kshitish K. Acharya

**Affiliations:** 1 Institute of Bioinformatics and Applied Biotechnology (IBAB), Bengaluru (Bangalore), Karnataka State, India; 2 Shodhaka Life Sciences Pvt. Ltd., Bengaluru (Bangalore), Karnataka State, India; Auburn University, United States of America

## Abstract

**Background:**

Gene expression profiling of uterus tissue has been performed in various contexts, but a significant amount of the data remains underutilized as it is not covered by the existing general resources.

**Methodology/Principal Findings:**

We curated 2254 datasets from 325 uterus related mass scale gene expression studies on human, mouse, rat, cow and pig species. We then computationally derived a ‘reliability score’ for each gene's expression status (transcribed/dormant), for each possible combination of conditions and locations, based on the extent of agreement or disagreement across datasets. The data and derived information has been compiled into the ***M*** *ammalian* ***G*** *ene* ***Ex*** *pression* ***U*** *terus* ***d*** *ata* ***b*** *ase* (MGEx-Udb, http://resource.ibab.ac.in/MGEx-Udb/). The database can be queried with gene names/IDs, sub-tissue locations, as well as various conditions such as the cervical cancer, endometrial cycles and disorders, and experimental treatments. Accordingly, the output would be a) transcribed and dormant genes listed for the queried condition/location, or b) expression profile of the gene of interest in various uterine conditions. The results also include the reliability score for the expression status of each gene. MGEx-Udb also provides information related to Gene Ontology annotations, protein-protein interactions, transcripts, promoters, and expression status by other sequencing techniques, and facilitates various other types of analysis of the individual genes or co-expressed gene clusters.

**Conclusions/Significance:**

In brief, MGEx-Udb enables easy cataloguing of co-expressed genes and also facilitates bio-marker discovery for various uterine conditions.

## Introduction

Uterus is an important mammalian organ that must be well studied for its role in normal functions such as sperm migration, embryo implantation and fetal nourishment, as well as multiple disorders [1], [2]. Cervical cancer is one of the leading causes of cancer deaths in women worldwide [3]. Similarly, endometrial cancer, endometriosis and infertility due to defective uterine functions have also been major human health concerns. Much remains unknown about the normal physiology and pathological details of the uterus tissue.

Understanding the pattern and mechanisms of regulation of gene expression is central to most aspects of biology, including the normal and abnormal states of the mammalian uterus. Large-scale detection of gene expression patterns is easier at the transcript level when compared to the protein level. Microarrays enabled genome wide transcript profiling and they have been used extensively to explore various biological phenomena.

Variations in the expression level and status of genes, across the results of microarray experiments [4], have caused limitations in the utilities of such gene expression data. Recommended standards for microarray experiments and reporting [5]–[7], and improved meta-analysis methods [8]–[11] might facilitate a better use of the reported data. While scientists today seem to prefer sequencing based methods for transcript profiling [12], [13], the value of the already existing microarray data cannot be underestimated. Microarray and other high-throughput gene expression data have been compiled into multiple useful databases/repositories (for a list, see http://www.startbioinfo.com/gene-expression). But the inefficiencies in search options specific to physiological and experimental conditions also limit the exploitation of the available databases. It has also been observed that a significant amount of the data is missing in such databases [14], [15]. Compiling most of the expression data in one place would be a huge challenge due to two main reasons: a) gathering the data scattered in literature is a laborious task, but there seems to be no alternative; b) there has not been a convenient means to derive usable information across different platforms, studies and data types (raw/processed data or the final calls only). Our team earlier spent around 3 years to painstakingly compile gene expression data for the mammalian testis, and then applied the novel consensus based reliability assessment method to derive a binary expression status for each gene [15].

A similar effort is required for the uterus tissue. Significant amount of microarray data is indeed available for the mammalian uterus tissue [16]. There have been a few databases specific to a component of uterus such as endometrial tissue, (Endometrial Data Base: http://www.endometrialdatabase.com and SCCPIR Endometrium Database Resource: http://endometrium.bcm.tmc.edu/edr) or to a condition, CCDB, Cervical Cancer gene DataBase [17]. But, there has not been a uterus specific database. With an intention to compile maximum existing uterine gene expression data and aid research on various aspects of mammalian uterus, we have created the **M**ammalian **G**ene **Ex**pression **U**terus **d**ata**b**ase (MGEx-Udb), and are reporting the same.

## Results

### Database content

**Figure 1:**
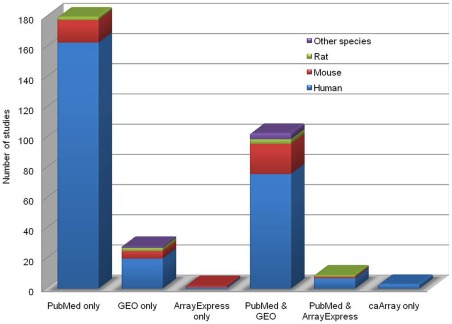
Source of data across various mammalian species in MGEx-Udb. Other species include cow and pig. Among the data collected from GEO or “PubMed & GEO”, 85% of the studies were also present in ArrayExpress, even though this is not indicated in the figure.

**Figure 2:**
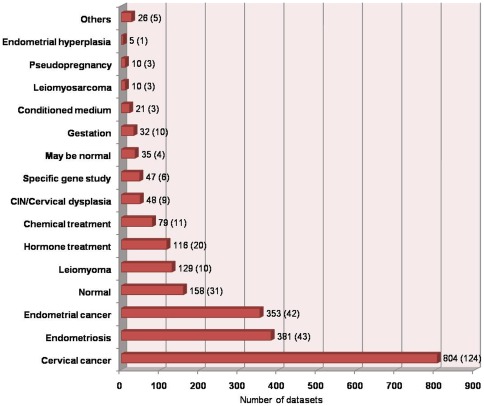
Number of datasets (**and studies**) **in MGEx-Udb corresponding to various physiological and pathological uterine conditions.** ‘Others’ represent post-parturition, genetic-ablation, artificial insemination and embryo implantation. Studies considering tissues that are used as controls but may not be absolutely ‘normal’ have been grouped in *‘may be normal’* category (examples: “normal tissue adjacent to tumor/cancer tissue”, “vehicle-treated”).

(A) Data considered for scoring: Currently, the database covers 325 studies with 2254 datasets corresponding to 1092 ‘Expression Status under specific Locations and Conditions’ (ESLCs) for human, mouse, rat, cow and pig. About 83% of the data in MGEx-Udb is from studies on human species (Figure 1). The database provides 970 different ESLCs for human (23,735 genes), 91 for mouse (24,428 genes), 15 for rat (14,497 genes), 8 for cow (10,875 genes), and 8 for pig (1,720 genes). The database has maximum number of studies for cervical cancer (38% of all studies). The next most abundant studies correspond to endometrial cancer and endometriosis (approximately 13% studies for each). Other contributing conditions include the normal, leiomyoma, leiomyosarcoma, cervical intraepithelial neoplasia (CIN), endometrial hyperplasia, endometrial cycles, gestation, treatment with chemicals/hormones and knockout and transfection studies associated with specific genes (Figure 2). Most disease related reports are from human tissues and/or cell lines. While studies related to hormone treatment, embryo implantation and normal tissue are common in mouse, studies on chemical/hormone treatment and endometriosis are common in rat. In case of cow and pig, pregnancy related studies are common.

**Figure 3:**
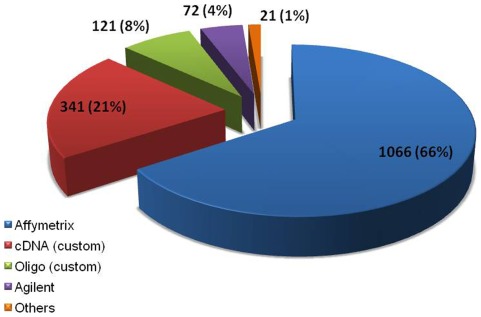
Datasets across various microarray platforms in MGEx-Udb. ‘Others’ include datasets contributed mainly by GE Healthcare and Illumina platforms.

**Figure 4:**
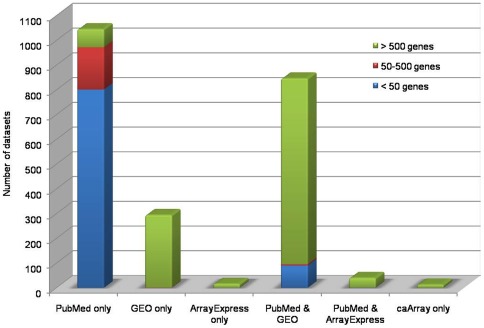
Datasets (**with gene count**) **collected from various sources.** In case of “PubMed & GEO” and “PubMed & ArrayExpress”, smaller gene lists came from validation experiments and were collected from PubMed, while raw/processed data were always collected from the repositories (GEO/ArrayExpress).

Of the 325 studies identified for data collection, 295 published research articles were curated to collect the information associated with each gene list. In remaining cases, the required information was curated directly from repositories; there was no corresponding publication for these experiments. Of all the studies, around 55% were exclusively obtained from literature. The remaining data came from Gene Expression Omnibus (GEO) [18], ArrayExpress [19] and other repositories alone, or in combination with literature (Figure 1). In the database, 90% of studies correspond to mRNA level expression and 10% studies correspond to expression at proteomic level. Most (91%) of the mRNA level reports came from microarray technology, which also contributes to 72% of the total datasets. Affymetrix (66%) is the leading contributor among the microarray platforms, followed by cDNA custom arrays (21%) (Figure 3). Small-scale studies based on reverse transcription Polymerase Chain Reaction (PCR), quantitative real time PCR, blotting techniques, etc., also contributed datasets. Among the total datasets, 52% have >500 genes in each, 8% of them have 50–500 and the remaining 40% contain <50 genes (Figure 4). In most cases, datasets corresponding to small-scale studies were from the validation experiments of a mass scale gene expression study.

B) Data not considered for scoring: MGEx-Udb also has sequencing data. Such data could not be employed in scoring the consensus due to the incompatibility of these data types with the current computational scoring system. Next Generation Sequencing (NGS) data was included for HeLa cells with differential expression calls for 2 treatment conditions, from 3 studies. Links are provided for other relevant NGS (raw) datasets. Bulk of the sequencing data, however, corresponds to Expressed Sequence Tags (ESTs).

**Figure 5:**
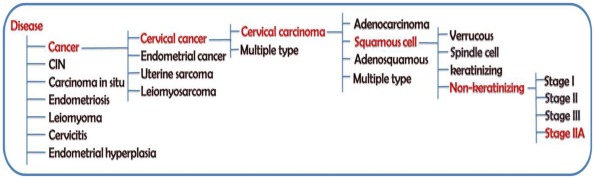
Example hierarchy of the conditions and sub-conditions. An example *(“stage IIA non-keratinizing squamous cell cervical carcinoma”)* hierarchy of the conditions and sub-conditions, for which data have been collected, and drop-down options provided in the query and upload pages of MGEx-Udb. Currently the database allows up to four levels of the hierarchy to query.

### Web interface

#### Query features

MGEx-Udb provides multiple query options. To query by a gene, user can enter identifiers of one of the following types: names, symbols, synonyms, Entrez gene IDs, and gene keywords/descriptions. Condition-based search can be done by selecting the condition of interest from the drop-down options of physiological or experimental conditions at different levels of hierarchies for a chosen species. For example, genes transcribed or dormant can be obtained for human cervical cancer as well as, squamous cell carcinoma condition. Similarly, queries can be restricted to a specific region of the tissue (sub-tissue), and cell-type. There is also an option to choose a specific population type such as Caucasian, in case of humans, and strain types such as C57BL6 or Sprague-Dawley in case of mouse and rat, respectively.

#### Output

For gene-based search, the database provides a list of identical as well as partially matching genes in different species. Each gene in this page can be clicked for basic information on the gene, their promoters, expression status, products (transcripts and proteins), Gene Ontology (GO) annotations, protein-protein interactions, cross-reference to other major bioinformatics resources, and relevant PubMed citations. Basic gene information consists of sequence, loci and gene summary. Transcript information includes transcript ID, coding sequence and exon-intron details. Promoter details cover the Transcription Start Site (TSS), potential promoter sequence and its chromosomal position. Protein information provides different isoforms of the protein(s), with corresponding sequence, function, molecular weight and amino acid length. Expression status(s) of the gene is shown, along with a reliability score, for multiple tissue regions/sub-tissue and cell-types under different physiological and experimental conditions. The original source of the expression data is displayed in a separate panel. In addition to this, the database shows an indicative expression status(s) based on sequencing data (EST & NGS), for various uterine tissues/conditions.

Query with a condition (condition-based search) provides lists of genes transcribed and dormant in the queried condition. In each of these two lists, the ‘reliability score’ is shown for every gene. In fact, the genes are arranged in the descending order of their scores. GO annotations are also displayed for the first 100 genes. The user can export the complete list of genes along with their reliability scores. References to the source datasets considered for scoring can be viewed in this output page. Clicking on any gene in the result page will be similar in effect to the gene specific query described in the previous paragraph. The ‘analyze’ option in the output page allows the user to perform quick analysis of significant functions/processes of the selected genes. The user can quickly initiate GO analysis and multiple sequence alignment (of genes, proteins and promoters), and easily access relevant pathways and Single Nucleotide Polymorphism (SNP) records. The database also permits co-expression, protein-interaction and pathway analyses, and offers visualization of the networks among the selected cluster of genes using GeneMANIA [20] functional analysis tool.

The database includes easy browsing of genes and conditions. In addition, links are provided to uterus related data (NGS and copy number variations), with an index of conditions, and other resources.

## Discussion

A significant amount of the published microarray data is not found in any of the widely used databases or repositories [14], [15]. Compilation of such data has to be manual and would be a time taking process. We have initiated tissue wise compilation of mammalian gene expression data with an aim to use the existing data for cataloguing the gene expression patterns. A comparative study [15] of the databases with condition specific queries indicated the superiority of such tissue wise biocuration of the gene expression data. A similar comparison of MGEx-Udb with other repositories/databases showed that former provides easier query system and provides higher number of relevant studies and genes (details in statistics section of the database).

The strength of the ‘reliability score’, for the binary expression status, is proportional to the amount of datasets and agreement across them, for any corresponding condition. There are some limitations [15] in such consensus based scoring of the binary expression states. But, this binary consensus method does offer a significant advantage over most other meta-analysis methods in deriving a semi-quantitative consensus. It works across platforms and technologies, irrespective of availability of raw/processed data as long as the final call has been made.

The hierarchical display of genes transcribed/dormant in specific conditions can be a useful representation of the transcription profiles. The higher scores indicate consistency in expression status of the corresponding genes across biological samples (used in different studies) and technologies. In fact, the consistency seems to be maintained for many genes despite the variations in the technology such as the microarray platform, RNA isolation methods and statistics, as well as the samples, which could also vary in terms of populations/strains and other related aspects such as age, social interactions and diet. The resulting lists can be used to identify genes that have strong association with any physiological status/condition in mammalian uterus tissue. For example, user can obtain a list of genes that are transcribed or dormant in the disease condition of interest and compare with those having the opposite expression status in the normal condition. A union list of genes across the two conditions can be derived and hierarchically arranged based on scores. Such a list would include genes with varying degrees of association with the disease. As an example, the genes *“transcribed in cervical cancer but dormant in normal cervix/uterus”* with high reliability scores may be better candidate bio-markers than the genes usually identified as differentially expressed by a single study. *CDKN2A*, which is an already well-known marker for cervical cancer [21], [22], is one such gene that has a score of 318 for *‘transcribed status in cervical cancer’* from 79 studies from PubMed, 6 from GEO, 1 from ArrayExpress and 2 from caArray, and 88 for *‘dormant status in normal uterus’,* from 32 studies from PubMed and 1 from GEO. On the contrary, some of the top genes from the dormant list for cervical cancer were also dormant in normal uterus, and hence they are less likely to have a strong association with the disease. Thus, the output obtained across conditions can be used to differentiate genes that have strong association with a uterine condition from those with weak or no association. This approach could pave a new way of listing potential diagnostic, prognostic and therapeutic targets for the uterus related disorders. This process can be used to obtain refined clusters of co-expressed genes.

The gene clusters obtained by MGEx-Udb can be useful not only to understand the molecular mechanisms and pathways associated, but also to elucidate the mechanisms of transcriptional regulation, disease-stage identification, gene prioritization and gene function predictions. We have initiated some studies in the promoter analysis of some of the important clusters of co-expressed genes. The compiled (after a huge screening effort) list of references of the gene lists corresponding to each condition and location of interest can be particularly useful for users interested in applying other meta-analysis methods to the gene expression data.

Since MGEx-Udb provides most or all of the genes associated with a specific condition, it may serve as a good starting point for any kind of functional analysis for various uterine conditions. MGEx-Udb also provides an opportunity to compare gene expression patterns across subtle variations in conditions and treatments. For example, one can compare expression status from untreated normal tissue reports with those from sham/vehicle-treated samples (may be normal); tumor-adjacent tissues (may be normal) with tumor-lacking tissues (normal); uterine layers/cycles; stages of cancer etc - across studies.

The already existing resources that correspond to specific uterine sub-tissues/conditions are advantageous in some ways compared to MGEx-Udb. Hence, we have included links to such resources in our database. CCDB [17], a database specific to cervical cancer, not only provides up/down regulated, methylated, mutated and amplified genes, but also gives information on miRNAs related to cervical cancer. Endometrial Data Base (http://www.endometrialdatabase.com) and the SCCPIR Endometrium Database Resource (http://endometrium.bcm.tmc.edu/edr) compile several reports of differential gene expressions in endometrial conditions. But, apart from being limited to specific conditions, their gene coverage does seem to be less than MGEx-Udb. They are also not designed to provide a consensus expression status through meta-analysis, or facilitate such process. On the other hand, tissue specific databases such as TiGER [23] and TiSGeD [24] provide uterus specific genes, but do not allow specific queries for diseases and/or experimental conditions.

### Future developments

The current work has taken us 3 years, mainly due to the manual curation tasks involved. Data corresponding to some conditions and species is yet to be included and the scoring for binary status has its limitations. We intend to update the database with data for more mammalian species and uterine conditions by: a) inviting fellow scientists to upload the data, and b) our own efforts following additional funding. We are also planning to improve the scoring system in many ways: a) different weight assignment based on the number of samples, hybridizations and validation experiments; b) incorporate consensus on the differential expression status along with transcribed/dormant status; c) perhaps in collaboration with other organizations, establish methods to incorporate data from other high-throughput gene expression data, such as NGS and EST, while deriving the consensus.

### Summary

The newly developed MGEx-Udb is intended to boost multiple types of efforts by biologists working on the uterus tissue. The important applications/features of this database are the following. **A**) It includes a large amount of manually compiled gene expression data corresponding to uterus from various reports and databases. **B**) It provides a catalogue of co-expressed genes in various normal and abnormal uterine conditions. **C**) It provides a “reliability score” to indicate the extent of agreement or contradictions of the expression status across microarray and proteomic studies pertaining to a specific condition/cell-type, for each gene. **D**) It also uses sequencing data in various uterine tissues/conditions to indicate expression status of each gene. **E**) It can be queried with normal or any of the pathological conditions in uterus, as well as the genes, of mouse, rat and human species. **F**) In addition to the expression status along with reliability scores for multiple uterine conditions, the database provides easy access to other important basic details such as the sequences of the genes, proteins and transcripts, GO annotations, protein-protein interactions and the relevant citations. **G**) It allows performing sequence and functional analyses of the derived co-expressed sets of clusters. **H**) Every gene is also cross-referenced to other useful bioinformatics resources. **I**) It provides an easy access to the compiled list of references of gene lists corresponding to various uterine conditions, useful for various meta-analysis approaches. All these features are likely to catalyze the process of transcript cataloguing, and various other uterus related research efforts.

**Figure 6:**
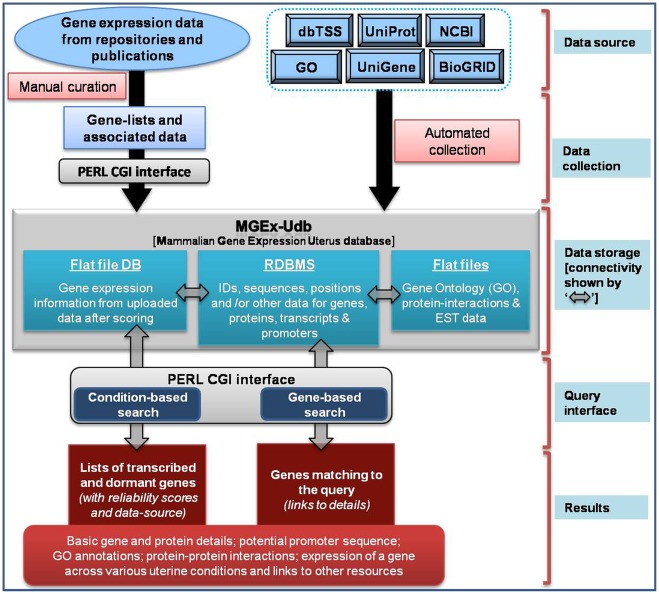
Schematic representation of MGEx-Udb. The figure represents the data collection (top portion), architecture (central portion) and operation (bottom portion) of the database.

## Materials and Methods

### Data collection

A search strategy was carefully designed to collect relevant articles reported in the literature, (detailed procedure can be found at http://dx.doi.org/10.1038/npre.2011.2101.3). Briefly, this involved identifying combinations of query terms/phrases for each search tool, obtaining the citations using multiple tools and then compiling the hits into a non-redundant union list using the Citation-Compiler tool (http://www.shodhaka.com/compiler). An example of the complete search strategy and query sets can be found in the FAQs section of the database. The aim was to collect citations related to mass scale gene expression in uterus tissue. An initial screening of the articles was performed to verify the relevance, by reading the abstracts. The articles identified as relevant were then searched for the list of genes reported to be expressed, up-regulated, down-regulated, etc by a thorough full text reading. Gene lists from these relevant articles were collected from manuscript, supplementary notes or the authors' website. Major repositories such as the GEO [18] and ArrayExpress [19], and other repositories such as Oncomine [25], Stanford Microarray Database (SMD) [26], Center for Information Biology gene EXpression database (CIBEX) [27], caArray (https://array.nci.nih.gov/caarray), GEMMA (http://www.chibi.ubc.ca/Gemma/) and Public Expression Profiling Resource (PEPR) [28] were also searched for the large scale gene expression data pertaining to mammalian uterus tissue. Processed data was collected wherever available, as the scoring method only requires the final call about the present/absent status of the genes. If there was no processed data, raw data was downloaded and processed using suitable standard methods as recommended in the Bioconductor packages (http://www.bioconductor.org).

Along with the gene list, associated information such as the expression status, species, tissue and sub-tissue or cell line, cell-type, and the corresponding physiological or experimental conditions were collected from the publications or repositories. This set of basic parameters is henceforth referred to as ‘Expression Status under specific Location and Condition’ (ESLC). The ‘conditions’ include normal physiological state, diseases, endometrial cycles, gestation, treatment with hormones and/or other chemicals, etc. A controlled vocabulary was set for each condition, to maintain the uniformity and to derive the consensus across similar studies. Figure 5 illustrates the hierarchy of a condition and multi-level sub-conditions. Other information collected about the gene lists included the number of samples, age of individuals, number of RNA isolations and hybridizations, and the details of main as well as validation experiments (example: platform, probes and statistical methods). These gene lists along with the annotated information (henceforth referred to as datasets) were uploaded to the database. The minimum number of genes per dataset was 3, the maximum was 21609, and the average was 8554. Every entry was cross-checked by at least one other researcher and, on an average 0.7% errors (e.g., gene chip name, population type, time course of treatment) were detected and rectified.

A ‘reliability score’ was derived for every ESLC of each gene, using procedures described earlier [15], to indicate the extent of agreement or disagreement across datasets, which correspond to same or similar conditions and locations for each species. Higher scores indicate that the corresponding genes are consistently reported to be transcribed or dormant. Genes with lower scores for the same/similar conditions would indicate either lesser number of corresponding studies or presence of contradicting reports for the specific expression status under consideration.

Sequencing data related to uterine tissues/conditions was also compiled. While reports on RNA sequencing were collected from literature, EST data was directly taken from UniGene [29].

### Database creation

Perl based CGI script was used to create an interface for entry of gene lists and associated information. An in-house database was used to convert the gene identifiers from the datasets into Entrez gene identifiers. These Entrez gene identifiers were queued-up for downloading other gene related information. LWP module (http://search.cpan.org/~gaas/libwww-perl-5.836/lib/LWP.pm) was used to connect to NCBI and the required information was downloaded with the aid of NCBI E-utilities (http://eutils.ncbi.nlm.nih.gov/entrez/query/static/eutils_help.html). Downloaded information includes official gene symbol, aliases, gene sequence, gene summary, chromosomal location, potential promoter sequence [−1000 to +200 bp] and all transcript sequences (along with exon-intron details) corresponding to each gene. Protein related information was downloaded from UniProt (http://www.uniprot.org; [30]). Similarly, transcription start sites were downloaded from dbTSS (ftp://ftp.hgc.jp/pub/hgc/db/dbtss/; [31]), version 7.0. When the information was not available in dbTSS for a gene, the 5′ end of corresponding NCBI gene sequence was used to represent the TSS position. Gene Ontology information was downloaded from the ftp site of the database (ftp://ftp.geneontology.org/pub/go/; [32]) and protein-protein interaction information was downloaded from BioGRID (http://thebiogrid.org/download.php; [33]), version 3.1. EST data was downloaded from UniGene (ftp://ftp.ncbi.nih.gov/repository/UniGene; [29]). Perl codes were written to ensure automatic incorporation of the downloaded data into the database. ClustalW was downloaded from http://www.clustal.org/clustal2/ and integrated into database, to provide facility to perform multiple sequence analysis.

MySQL Relational Database Management System (RDBMS) is used for storing data. A table is dedicated to store the basic gene related information including the gene name, locus and transcript details. Another table is used to store gene identifiers such as the gene name, gene description, official gene symbol and the NCBI gene identifier, microarray platform probe identifiers, etc. Separate tables are maintained to store information related to species, cell-type, tissue, cell line and conditions which make up ESLC. Each entry in these non-redundant tables is tagged with unique identifier. The results obtained from scoring system are maintained as flat file database. Each file corresponds to unique ESLC, which is named using identifiers from ESLC tables. The complete database architecture and function is represented in a schematic in Figure 6.
